# Investigation into the Reprocessability of Polycarbonate/Organoclay Nanocomposites

**DOI:** 10.3390/polym18010067

**Published:** 2025-12-26

**Authors:** Basak Tuna

**Affiliations:** Department of Metallurgical and Materials Engineering, Kirsehir Ahi Evran University, Kirsehir 40100, Turkey; basaktuna@ahievran.edu.tr

**Keywords:** polycarbonate, organoclay, reprocessability, degradation, extrusion

## Abstract

With the rapid expansion in the use of nanomaterials, ensuring their reprocessability has become a critical consideration for the sustainable development of polymer-based nanocomposites. In this study, the effects of repetitive thermo-mechanical processing cycles on the properties of polycarbonate (PC)/organoclay nanocomposites, as well as the impact of reactive extrusion of reprocessed PC/organoclay nanocomposites using a chain extender, were investigated for the first time. The nanocomposites were processed three times using a twin-screw extruder, and a multi-anhydride functional chain extender was incorporated to counteract the thermo-mechanical degradation observed after the third extrusion cycle. Morphological analysis indicated that the delamination of clay nanolayers within the polymer matrix was slightly enhanced with increasing extrusion cycles, while the addition of the chain extender further promoted nanoclay exfoliation. Despite the improved clay dispersion in PC, both rheological and tensile measurements revealed the detrimental effects of repeated reprocessing on the nanocomposites. The chain extender effectively mitigated this degradation by relinking cleaved polymer chains; consequently, the complex viscosity and storage modulus at 0.1 Hz of the three-times-extruded nanocomposite increased by 248% and 426%, respectively, following chain extender incorporation. The effectiveness of the chain extender was further evidenced by a 27% enhancement in tensile strength. The glass transition temperatures of the samples were not significantly affected by either the extrusion cycles or the addition of the chain extender. The thermal stability of the nanocomposites decreased with increasing numbers of extrusion cycles; however, the incorporation of the chain extender imparted enhanced resistance to thermal degradation, as confirmed by thermogravimetric analysis.

## 1. Introduction

The consumption of plastics has increased dramatically over the past decade owing to rapid population growth, urbanization, and economic development [1]. Global plastic consumption is projected to reach 884 Mt by 2050, nearly double the 464 Mt reported in 2020 [2]. Innovations in materials science have driven the development of advanced materials capable of surpassing traditional metals, ceramics, and conventional plastics. With advancements in nanotechnology, interest in nanocomposite materials has grown significantly, enabling the design of materials with enhanced and multifunctional properties. Polymer-based nanocomposites have attracted considerable attention due to their ease of processability, low density, and relatively low cost compared with materials such as metals and ceramics. Polymer nanocomposites are hybrid materials in which at least one dimension of the dispersed phase is in the nanometer scale, and they are utilized in a wide range of engineering applications, including defense, packaging, transportation, electrical and electronic systems, and energy technologies [3].

In response to the increasing industrial demand for polymers and their corresponding nanocomposites, the recycling of plastic waste has become critically important from environmental, social, and economic perspectives. Although several recycling approaches, such as chemical recycling and incineration, are available for polymer waste, mechanical recycling through injection molding or extrusion is a preferred method because it does not require solvents or generate toxic gaseous byproducts, and can be readily implemented in industry in a cost-effective manner [4,5].

Polycarbonate (PC) is an amorphous engineering thermoplastic that exhibits high tensile strength, good processability, chemical resistance, and optical clarity, making it widely used in industries such as automotive, construction, and electronics [6,7]. The development of PC/nanoclay nanocomposites represents a practical approach to further enhancing the performance of PC for broader applications [8]. The principal methods for preparing polymer–nanoclay nanocomposites include solution mixing, in situ polymerization, and melt compounding. Among these, melt compounding is more environmentally benign and industrially viable, as it promotes effective delamination of clay layers within the polymer matrix through high shear forces [9]. However, a limitation of this method is the thermal degradation of nanoclays at elevated processing temperatures, particularly for high-melting polymers such as PC [10]. The modification of hydrophilic nanoclays with organic cations to form organoclays is therefore essential for their compatibility with organic polymers [11]. Quaternary ammonium salts are commonly employed as organic modifiers in commercial nanoclays; however, they undergo thermal degradation via the Hofmann elimination reaction at temperatures above 180 °C [12]. This reaction generates Brønsted acidic sites, which accelerate polymer degradation. The primary degradation mechanisms of PC involve scission of isopropylidene or carbonate linkages, producing phenolic compounds with hydroxyl end groups [13]. Such chain scission results in deterioration of the thermal stability, viscosity, and molecular weight of PC [14,15,16,17,18].

As the applications of PC/clay nanocomposites continue to expand, the reclamation and recycling of these materials have become increasingly important. In mechanical recycling of polymer–clay nanocomposites, it is essential to investigate the effects of repeated melt-processing cycles on material properties. This evaluation enables identification of the reprocessing threshold at which property degradation due to organoclay-induced matrix degradation outweighs any potential property enhancement arising from nanoclay exfoliation or intercalation. These competing effects may occur simultaneously, with one dominating depending on processing conditions. Although the influence of reprocessing cycles has been extensively studied for various polymer–clay nanocomposites, such investigations have not extended to PC/clay nanocomposites [19,20,21,22].

The detrimental effects of reprocessing cycles on the properties of neat PC have been reported in the literature [23,24,25]. Consequently, strategies for restoring molecular weight and improving the properties of reprocessed PC have attracted significant interest. One promising approach is reactive extrusion using chain extenders at low concentrations (≈1 wt.%), which can be directly incorporated during melt processing without the need for additional equipment. Although the application of reactive extrusion with chain extenders to recycled PC remains limited, existing studies clearly demonstrate the effectiveness of this strategy in upgrading the performance of recycled PC. Villalobos et al. (2006) [26] investigated the efficiency of a multi-epoxy-functional chain extender in restoring the molecular weight of three recycled PC grades, reporting approximately 95–300% increases in molecular weight at a loading of only 1 wt.% during twin-screw extrusion. In a more recent study, Tuna (2023) [27] evaluated the performance of two chain extenders—one with multi-epoxy functionalities and another with multi-anhydride functional groups—in recycled PC. Both chain extenders improved the thermal stability, rheological behavior, and mechanical properties of recycled PC when added at 1 wt.% during extrusion. However, the anhydride-based chain extender exhibited superior efficiency, yielding a 58% increase in complex viscosity at 1 Hz compared to a 27% increase achieved with the epoxy-based chain extender. It was concluded that the use of anhydride chain extenders, which interact with terminal hydroxyl groups of phenolic compounds in recycled PC, represents a more effective approach. These findings highlight the potential of multi-anhydride-functional chain extenders to upgrade the properties of reprocessed PC/nanoclay nanocomposites, which have not been previously explored.

The objective of the present study is to investigate the effects of repeated processing cycles on PC/organoclay nanocomposites and to restore the properties of reprocessed nanocomposites through reactive extrusion. Accordingly, the nanocomposites were subjected to three sequential extrusion cycles, and a multi-anhydride-functional chain extender was incorporated into the three-times-extruded nanocomposite to examine its influence on the morphological, rheological, mechanical, and thermal properties.

## 2. Materials and Methods

### 2.1. Materials

The polymer matrix used in this study, polycarbonate (PC, Makrolon^®^ 2605), was purchased from Covestro, Leverkusen, Germany. To avoid additional chemical modification steps or the use of compatibilizers to enhance interactions between PC and nanoclay, Cloisite^®^ 30B (C30B) supplied by Southern Clay Products Inc, Gonzales, TX, USA. was selected as the organoclay for the experimental work. C30B is a montmorillonite clay modified via a cation-exchange process using methyl, tallow, bis(2-hydroxyethyl) quaternary ammonium ions. The hydroxyl moieties of this organoclay and the carbonyl groups of PC can form hydrogen bonds during melt compounding, thereby enhancing interfacial interactions between the polymer matrix and the nanoclay [28]. The chain extender, commercially known as Joncryl^®^ ADR 3400 (J), was kindly provided by BASF, Ludwigshafen, Germany. This chain extender contains anhydride functionalities capable of reacting with the chain-end groups of recycled PC, according to reaction mechanism presented by work of Tuna (2023) [27].

### 2.2. Sample Preparation

Prior to melt compounding, PC, nanoclay, and the chain extender were dried under vacuum at 90 °C for 12 h to remove residual moisture. Neat PC and PC containing 4 wt.% nanoclay were compounded at 260 °C for 4 min using a co-rotating laboratory-scale twin-screw compounder (Haake Minilab II micro-compounder, Thermo Fisher Scientific, Karlsruhe, Germany). Based on previous studies reporting enhanced properties of PC/nanoclay nanocomposites with increasing nanoclay content, a loading of 4 wt.% was selected, as it represents the highest nanoclay concentration that could be reliably processed using the compounder [14]. The compounding parameters were carefully optimized to promote uniform nanofiller dispersion while minimizing thermo-mechanical degradation of the polymer matrix. To evaluate the influence of multiple processing cycles, the extrudates of neat PC and the nanocomposite (NC) were collected from the die after each mixing cycle, pelletized, dried, and subsequently recompounded three times under identical conditions. To investigate the effect of the chain extender on the properties of reprocessed materials, samples subjected to three extrusion cycles were melt-mixed with 1 wt.% chain extender under the same processing conditions. The molten materials were then pelletized and dried following the procedure described above. The dried granules were subsequently compression molded at 260 °C under a pressure of 280 MPa for 5 min to prepare specimens for further characterization. Sample designations were assigned according to the number of extrusion cycles. For instance, NC-E3 denotes the nanocomposite subjected to three extrusion cycles, while its chain-extended counterpart is designated as NC-E3/J.

### 2.3. Characterization

Fourier transform infrared spectroscopy (FTIR) was conducted in attenuated total reflectance (ATR) mode over the wavenumber range of 650–4000 cm^−1^ using a PerkinElmer Spectrum 100 spectrometer (PerkinElmer, Waltham, MA, USA).

Morphological characterization was carried out using Zeiss Gemini 500 scanning electron microscope (SEM) (Zeiss, Oberkochen, Germany) and FEI Tecnai F30 transmission electron microscope (TEM) (FEI, Hillsboro, OR, USA). SEM images were obtained at an accelerating voltage of 3 kV, and fractured sample surfaces were sputter-coated with gold using Polaron SC500 (Fisons Instruments, Uckfield, UK) prior to imaging. TEM observations were performed on ultrathin sections prepared at −100 °C using a Leica EM UC6 ultramicrotome (Leica Microsystems, Vienna, Austria), with an accelerating voltage of 200 kV.

The rheological behavior of the specimens was analyzed using an Anton Paar Physica MCR 501 rotational rheometer (Anton Paar, Graz, Austria) equipped with parallel plates (25 mm diameter) and a gap of 1 mm, operated at 260 °C. Frequency sweep measurements were conducted over a range of 0.1–100 Hz at a strain amplitude of 0.4%, which was determined from preliminary amplitude sweep tests to ensure measurements within the linear viscoelastic region.

Mechanical properties were evaluated using an Instron 5564 universal testing machine (Instron, Norwood, MA, USA) at a crosshead speed of 5 mm/min in accordance with ISO 37 (Type 4) [29]. Three dumbbell-shaped specimens from each sample category were tested at room temperature.

Differential scanning calorimetry (DSC) measurements were performed using a PerkinElmer DSC 6000 (PerkinElmer, Waltham, MA, USA) under a nitrogen atmosphere. Sample masses were maintained below 10 mg and subjected to a heating cycle from 50 °C to 245 °C at 10 °C/min, followed by cooling to 50 °C at the same rate and a second heating to 245 °C. As the first heating cycle reflects the prior thermal history of the samples, data from the second heating cycle were used for analysis.

Thermogravimetric analysis (TGA) was carried out using a PerkinElmer Diamond thermal analyzer (PerkinElmer, Waltham, MA, USA) under a nitrogen atmosphere. Approximately 6 mg of each sample was heated from room temperature to 615 °C at a constant heating rate of 10 °C/min.

## 3. Results and Discussion

### 3.1. Fourier Transform Infrared Spectroscopy (FTIR)

**Figure 1:**
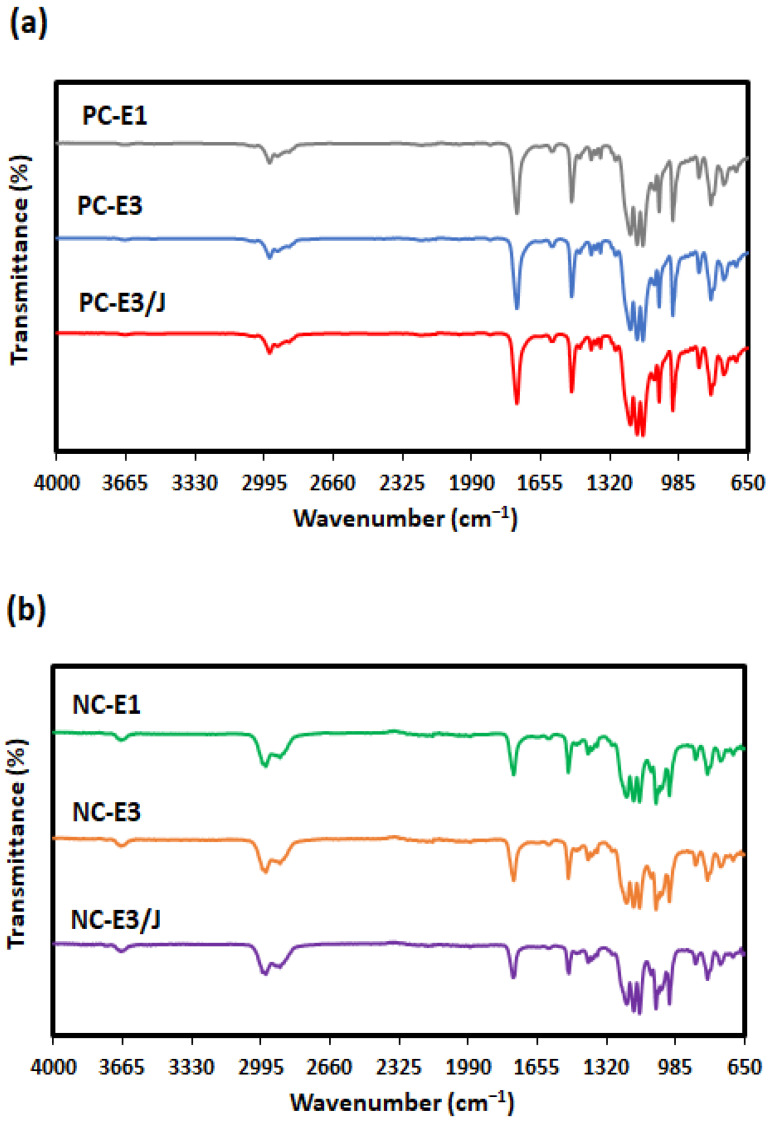
FTIR spectra of (**a**) neat PC and (**b**) PC/organoclay nanocomposites subjected to one- and three-times extrusion cycles with the third-cycle samples further modified using the chain extender.

FTIR spectroscopy is a widely used technique for identifying molecular structures and monitoring structural modifications in materials. The position and intensity of absorption bands provide critical insights into the composition and bonding characteristics of a material. During reprocessing, exposure to high shear forces and elevated temperatures may induce polymer chain scission and degradation. Conversely, reactions between the polymer and the chain extender may promote the reconnection of chain ends, thereby rebuilding the molecular network. Both degradation and chain-extension processes have the potential to modify the chemical structure of the polymer, and such changes may be reflected in the FTIR spectra. Nevertheless, as shown in Figure 1, the spectra of all samples remained essentially unchanged, indicating that neither reprocessing nor chain extension resulted in any significant alteration to the chemical structure of neat PC or the nanocomposites. Similar observations regarding the reprocessing of polymer nanocomposites and chain extender–nanocomposite systems have been reported in the literature [19,30,31].

### 3.2. Scanning Electron Microscopy (SEM)

**Figure 2:**
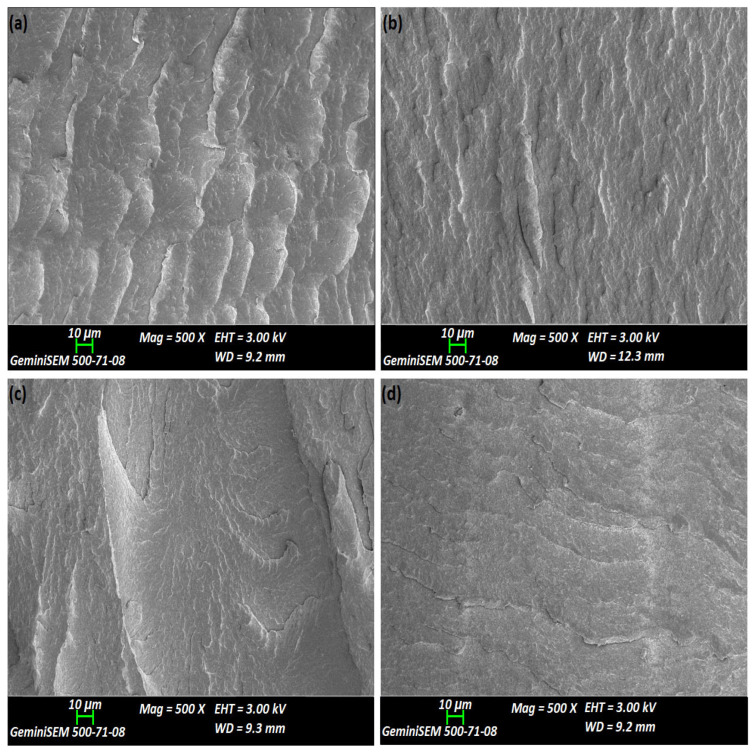
SEM images of nanocomposites: (**a**) NC-E1, (**b**) NC-E2, (**c**) NC-E3 and (**d**) NC-E3/J.

SEM was employed to evaluate the effects of reprocessing cycles and chain extender modification on the dispersion of nanoclay within the matrix at the micron scale. SEM images of the fractured surfaces of PC nanocomposites are presented in Figure 2. The chain-extended reprocessed nanocomposite shown in Figure 2d exhibits a smoother fracture surface, which can be attributed to the finer dispersion of clay particles. This behavior is likely associated with an increased molecular weight of the polymer matrix, which exerted stronger shear forces on larger clay particles during extrusion [32]. In contrast, the fractured surfaces of the reprocessed nanocomposites shown in Figure 2a–c display localized deformation, along with the presence of small clay agglomerates and microcracks within the matrix. These features may result from the increased coarseness of the fracture surfaces. A reduction in surface deformation and a lower number of agglomerates was observed with increasing numbers of reprocessing cycles, suggesting that repeated extrusion improved the delamination of clay nanolayers within the polymer matrix [33,34]. Since SEM could not provide information about exfoliation of nanoclays, more direct evidence was obtained through TEM analysis.

### 3.3. Transmission Electron Microscopy (TEM)

**Figure 3:**
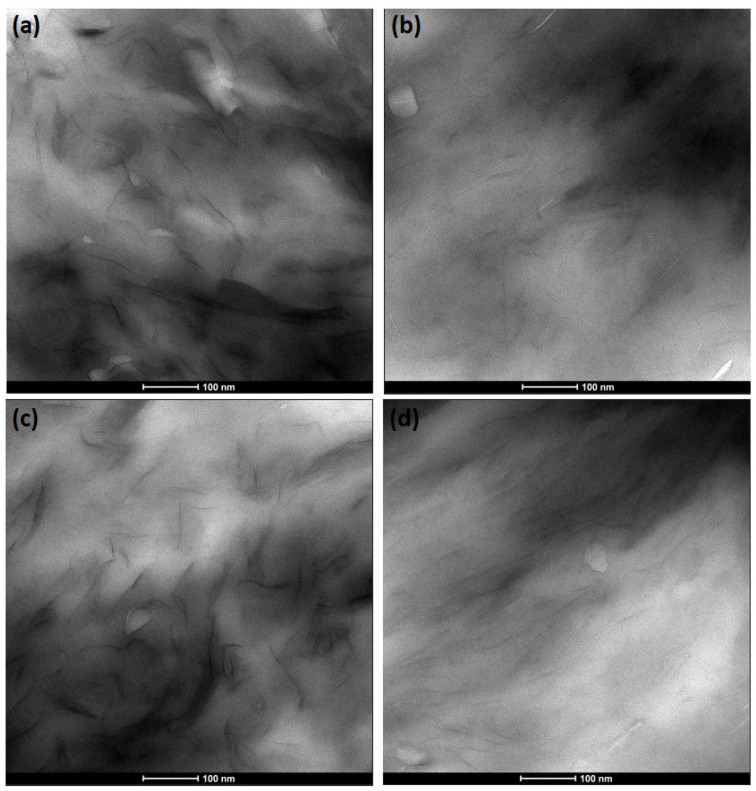
TEM images of nanocomposites: (**a**) NC-E1, (**b**) NC-E2, (**c**) NC-E3 and (**d**) NC-E3/J.

TEM was employed to evaluate the nanoscale dispersion state of clay within the nanocomposites. As shown in Figure 3, the nanocomposites exhibited finely distributed silicate layers within the PC matrix. This indicated that strong hydrogen bonding interactions between the hydroxyl groups of the organomodifier in the nanoclay and the carbonyl groups of PC facilitated the delamination of silicate layers during extrusion [28]. With respect to the effect of reprocessing, the TEM images (Figure 3a–c) corroborated the SEM observations, revealing that clay particles underwent extensive delamination with increasing reprocessing cycles. The morphology consisted predominantly of exfoliated platelets, along with intercalated tactoids composed of a limited number of nanolayers. Previous studies have emphasized that the generation of sufficient shear stress during melt compounding of polymer–silicate systems is critical for breaking down silicate stacks into smaller platelets, thereby promoting effective exfoliation [34,35]. Accordingly, the finer silicate dispersion observed after multiple reprocessing cycles can be attributed to intensified shear stresses that enhance dispersive mixing and inhibit re-agglomeration. The incorporation of the chain extender further promoted an exfoliated structure in the reprocessed nanocomposite, as evidenced by the TEM image in Figure 3d. These results are consistent with the SEM observations, which showed reduced clay particle sizes in the chain-extended reprocessed nanocomposite. The chain extension and branching reactions induced by the chain extender likely amplified the applied shear and elongational stresses on clay tactoids, resulting in their delamination into individual or few-layer silicate structures.

### 3.4. Rheology

**Figure 4:**
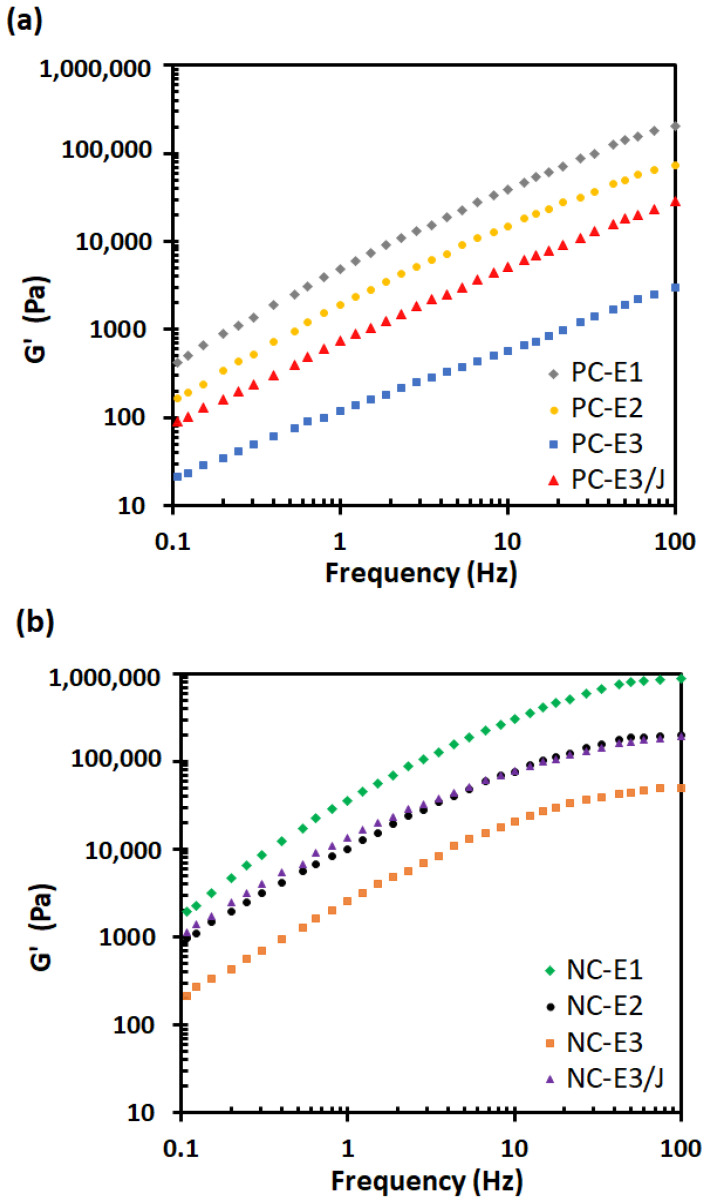
Elastic modulus (G′) vs. frequency for (**a**) neat PC and (**b**) PC/organoclay nanocomposites subjected to one, two- and three-times extrusion cycles with the third-cycle samples further modified using the chain extender.

**Figure 5:**
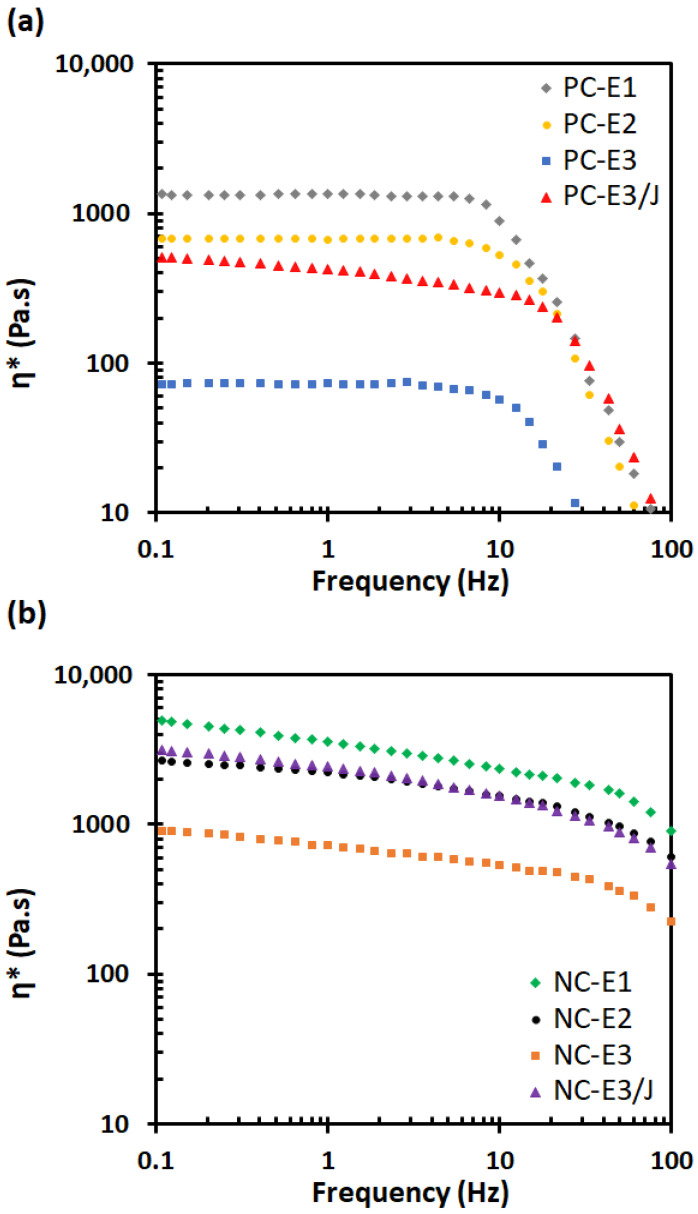
Complex viscosity (η*) vs. frequency for (**a**) neat PC and (**b**) PC/organoclay nanocomposites subjected to one, two- and three-times extrusion cycles with the third-cycle samples further modified using the chain extender.

Rheological analysis provides an effective means of distinguishing structural modifications induced by reprocessing, as it is highly sensitive to both the nanoscale organization of silicate layers and the viscoelastic response of the polymer matrix. Figure 4 and Figure 5 present the frequency dependence of the storage modulus (G′) and complex viscosity (η*) of the samples, respectively. As shown in Figure 4, a pronounced enhancement in the G′ of the nanocomposites was observed compared with neat PC, which can be attributed to favorable interfacial interactions between the organoclay and the polymer matrix. At a frequency of 0.1 Hz, for example, the G′ value of NC-E1 was 1927.2 Pa, whereas PC-E1 exhibited a value of only 428.7 Pa. The substantial increase in G′ at low frequencies in the presence of nanoclay suggests the formation of a percolated network structure, indicative of a higher degree of exfoliation and improved nanoclay dispersion within the polymer matrix, as corroborated by the SEM and TEM analyses.

Reprocessing cycles exerted a detrimental effect on the G′ of both neat PC and its nanocomposites. In the nanocomposite systems, repeated extrusion introduced two competing phenomena: polymer chain scission induced by thermo-mechanical degradation and enhanced nanoclay dispersion within the PC matrix [21]. The results indicate that chain scission was the dominant mechanism, leading to a pronounced decrease in G′ with increasing numbers of extrusion cycles. This behavior suggests that the ammonium-based organic modifier of the nanoclay accelerated PC degradation in the nanocomposites via the Hofmann elimination mechanism [36].

The incorporation of the chain extender significantly enhanced the G′ of both neat PC and nanocomposites subjected to three extrusion cycles. This improvement primarily resulted from the recoupling of degraded PC chains, along with branching reactions arising from the multi-anhydride functional groups of the chain extender. In the nanocomposite system, a modest increase in nanoclay exfoliation further strengthened polymer–filler interactions, thereby contributing to the observed enhancement in G′ [37].

As shown in Figure 5, the complex viscosity (η*) of all samples decreased with increasing frequency, indicating pronounced shear-thinning behavior characteristic of PC and its corresponding nanocomposites in the molten state. In the low-frequency region, the neat PC samples exhibited a clear Newtonian plateau (Figure 5a), whereas the PC/nanoclay nanocomposites displayed a more pronounced shear-thinning response (Figure 5b). This behavior can be attributed to the formation of a solid-like network structure arising from filler–filler interactions among well-dispersed nanoclay particles [28,38]. In addition, the viscosity of the PC/nanoclay nanocomposites was higher than that of neat PC, likely due to strong interfacial interactions, such as hydrogen bonding between the carbonyl groups of PC and the hydroxyl functionalities on the organoclay surface [28]. At 0.1 Hz, NC-E1 exhibited a complex viscosity of 4936 Pa·s, whereas PC-E1 showed a value of 1362 Pa·s.

Both neat PC and nanoclay-filled samples exhibited a decreasing trend in complex viscosity with increasing numbers of reprocessing cycles, indicating a reduction in molecular weight resulting from chain scission. At 0.1 Hz, the complex viscosity decreased from 1362 Pa·s for PC-E1 to 72.2 Pa·s for PC-E3. Although morphological observations revealed a slight improvement in nanoclay dispersion within the polymer matrix with repeated extrusion cycles, this enhancement was insufficient to compensate for the decline in complex viscosity of the nanocomposites. Specifically, NC-E1 exhibited a complex viscosity of 4936 Pa·s at 0.1 Hz, whereas NC-E3 displayed a markedly lower value of 911.8 Pa·s. These rheological results indicate that thermal degradation was the dominant phenomenon governing the reprocessing behavior of PC/organoclay nanocomposites and must be carefully considered during recycling.

Given the critical impact of thermal degradation on the reprocessability of these nanocomposites, the incorporation of a chain extender was investigated as a mitigation strategy. At 0.1 Hz, incorporation of the chain extender increased the complex viscosity of NC-E3 from 911.8 Pa·s to 3175.2 Pa·s, which was higher than that of NC-E2 (2666.6 Pa·s). This result demonstrates that the chain extender effectively reconnected cleaved PC chains, thereby increasing the molecular weight and restoring the melt viscosity.

### 3.5. Tensile Testing

**Figure 6:**
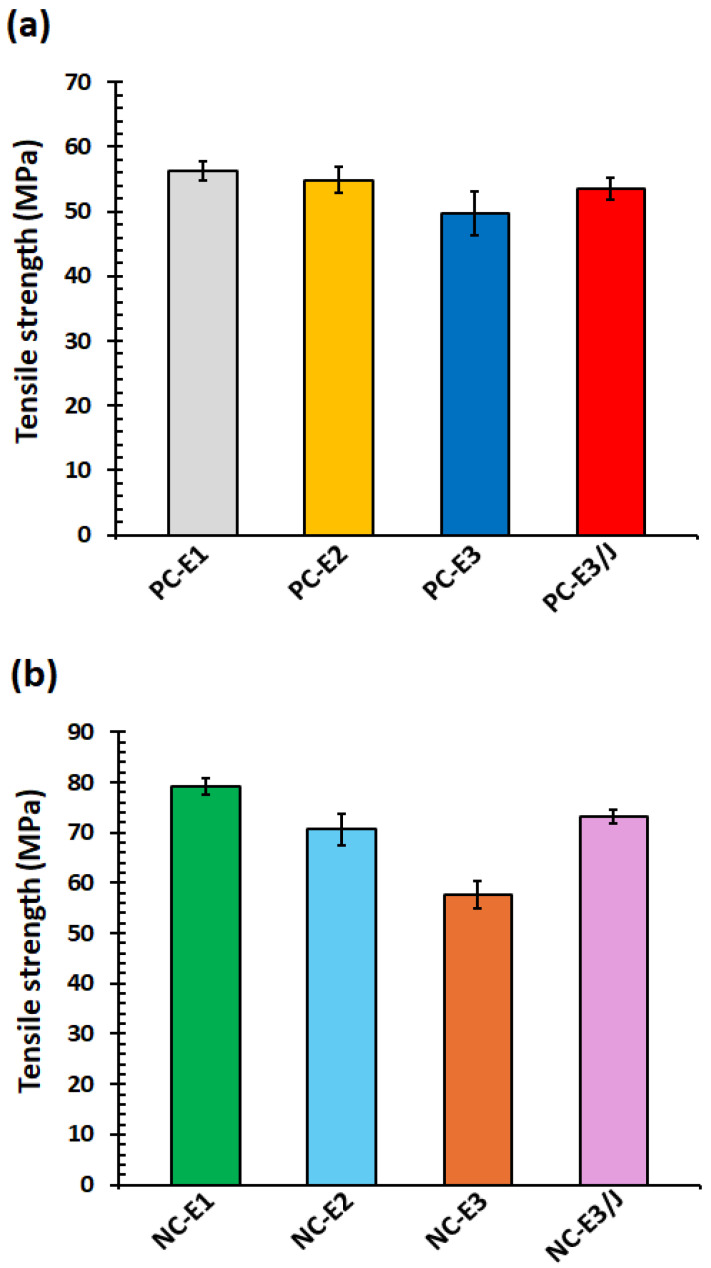
Tensile strength of (**a**) neat PC and (**b**) PC/organoclay nanocomposites subjected to one, two- and three-times extrusion cycles with the third-cycle samples further modified using the chain extender.

**Figure 7:**
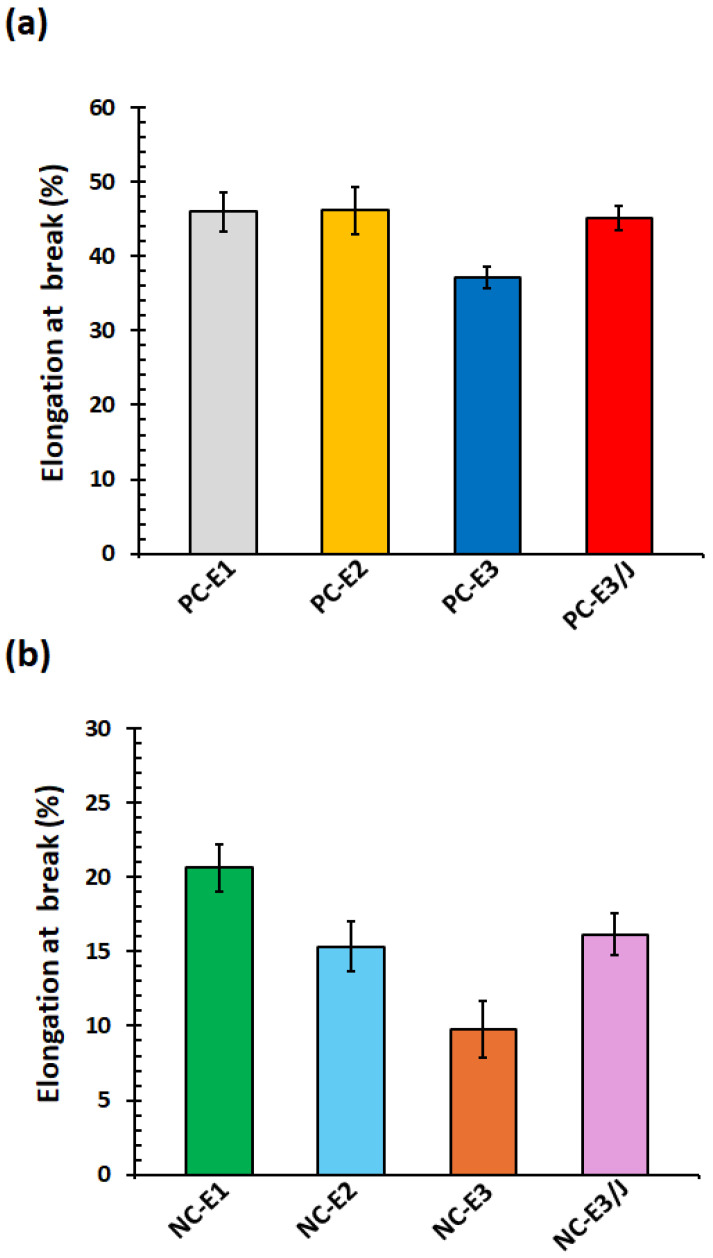
Elongation at break of (**a**) neat PC and (**b**) PC/organoclay nanocomposites subjected to one, two- and three-times extrusion cycles with the third-cycle samples further modified using the chain extender.

The tensile properties of polymer systems are influenced by several factors, including polymer–filler interactions and the molecular weight of the polymer matrix. The tensile test results for the samples are presented in Figure 6 and Figure 7. In particulate-filled composites, tensile strength primarily depends on the efficiency of stress transfer from the polymer matrix to the dispersed filler phase [39]. As shown in Figure 6, the nanocomposites exhibited higher tensile strength than neat PC, indicating effective stress transfer from the PC matrix to the nanoclay particles.

For neat PC, a continuous reduction in tensile strength with increasing numbers of extrusion cycles was observed, which can be attributed to molecular weight reduction caused by thermo-mechanical degradation [24]. Specifically, the tensile strength decreased from 56.2 MPa for PC-E1 to 49.7 MPa for PC-E3. Although a higher degree of nanoclay exfoliation is generally associated with enhanced mechanical properties in polymer nanocomposites, in the present study the degradation of PC during repeated extrusion dominated over any improvements arising from enhanced nanoclay dispersion [40]. Consequently, the tensile strength of the PC/nanoclay nanocomposites also decreased with increasing extrusion cycles, declining from 79.2 MPa for NC-E1 to 57.6 MPa for NC-E3.

The introduction of the chain extender to NC-E3 produced a dual beneficial effect. Recovery of molecular weight through chain-extension reaction, combined with improved nanoclay exfoliation, increased the tensile strength of the nanocomposite from 57.6 MPa for NC-E3 to 73.2 MPa for NC-E3/J.

The elongation at break of the samples exhibited two distinct behaviors, indicating a transition from ductile to brittle responses, as shown in Figure 7a,b. Consistent with previous reports, the incorporation of nanoclay substantially reduced the ductility of PC, as reflected by the decreased elongation at break [41]. The observed brittle behavior of the PC/nanoclay nanocomposites may be attributed to constraints imposed by exfoliated clay platelets and/or defect-like features associated with clay agglomerates [42].

Repeated extrusion cycles led to a slight reduction in elongation at break for both neat PC and the nanocomposites, which can be attributed to polymer chain scission [43]. In contrast, the incorporation of the chain extender led to a modest recovery in elongation at break. Specifically, PC-E3/J and NC-E3/J exhibited higher elongation at break values of 45.2% and 16.2%, respectively, compared with those of PC-E3 (37.1%) and NC-E3 (9.8%). This improvement is attributed to the formation of extended and branched polymer chains, which enhance chain entanglement within the samples and thereby increase their resistance to mechanical deformation [32].

### 3.6. Differential Scanning Calorimetry (DSC)

**Figure 8:**
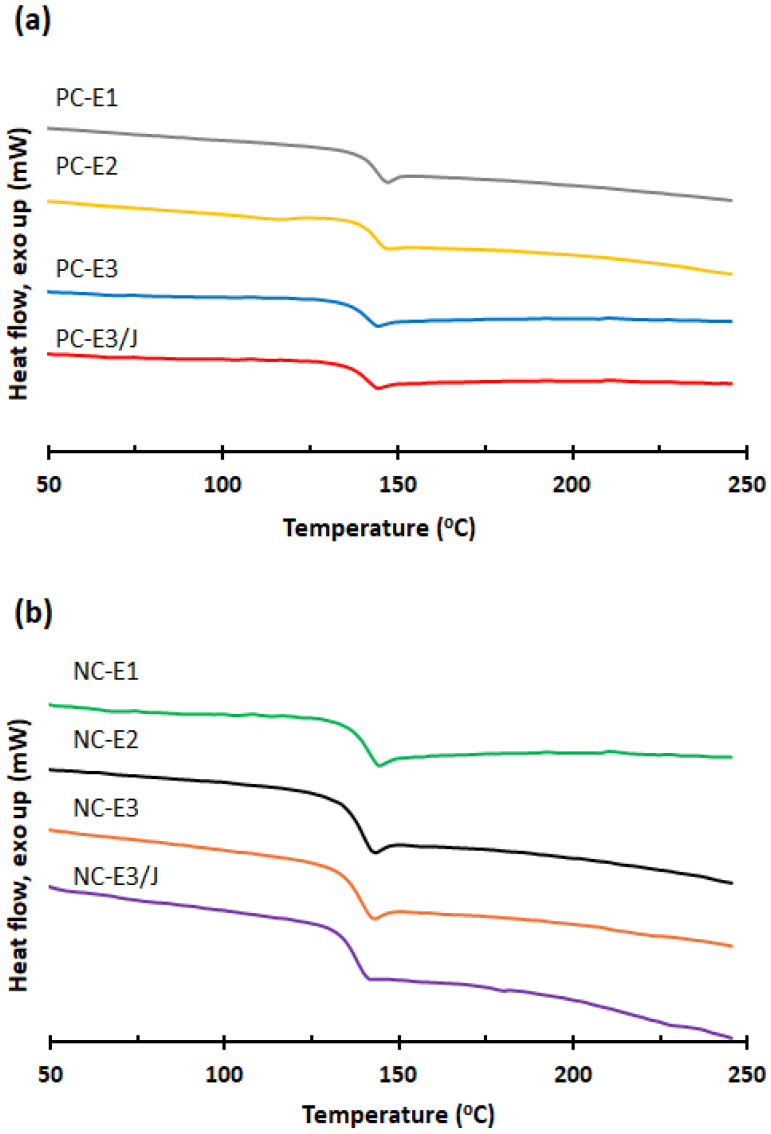
DSC thermograms of (**a**) neat PC and (**b**) PC/organoclay nanocomposites subjected to one, two- and three-times extrusion cycles with the third-cycle samples further modified using the chain extender.

The DSC thermograms of the samples are presented in Figure 8. As summarized in Table 1, all samples exhibited a single second-order transition corresponding to the glass transition temperature (T_g_), which is characteristic of amorphous polymers such as PC. For both unfilled and nanoclay-reinforced PC systems, T_g_ values decreased slightly with increasing number of reprocessing cycles, primarily due to the reduction in molecular weight observed during reprocessing. The PC/nanoclay nanocomposites exhibited lower T_g_ values than the neat PC samples, which can be attributed to the plasticizing effect of the nanoclay [17]. The limited effect of the chain extender on the T_g_ of the reprocessed samples is consistent with previous studies, which similarly reported that chain-extension reactions exert little effect on the glass transition behavior of recycled polymer systems [44,45].

### 3.7. Thermogravimetric Analysis (TGA)

**Figure 9:**
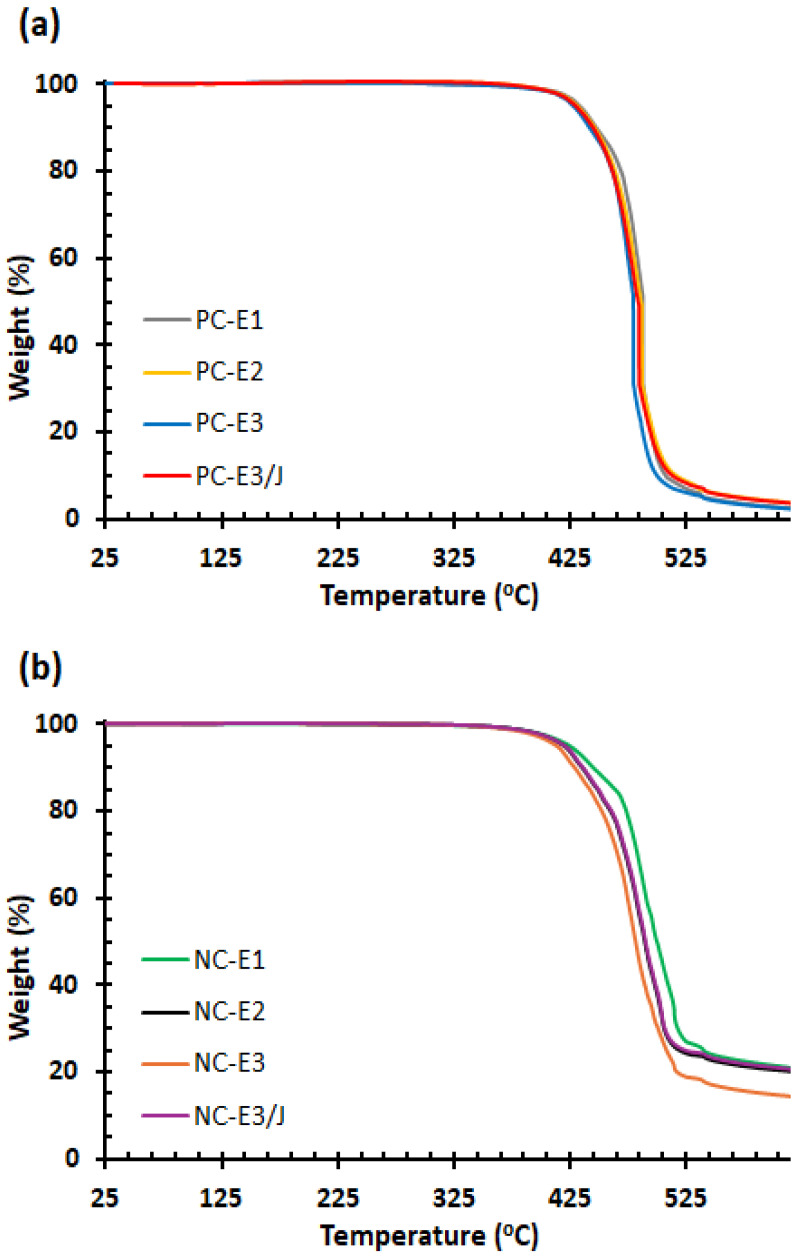
TGA curves of (**a**) neat PC and (**b**) PC/organoclay nanocomposites subjected to one, two- and three-times extrusion cycles with the third-cycle samples further modified using the chain extender.

The thermal stability of the samples was evaluated by using TGA. As shown in Figure 9 and summarized in Table 1, the thermal degradation temperatures corresponding to 5% and 10% weight loss (T_d5_ and T_d10_) of neat PC progressively decreased with repeated melt-processing cycles. This reduction is attributed to thermo-mechanical degradation during extrusion, which induces chain scission of carbonate ester linkages and the formation of phenolic compounds, thereby diminishing the thermal resistance of the polymer [18,27]. Specifically, the T_d5_ of PC-E1 decreased from 433.5 °C to 428.1 °C for PC-E3. The incorporation of the chain extender into PC-E3 improved its thermal stability, increasing T_d5_ to 430.3 °C for PC-E3/J, due to recoupling reactions between hydroxyl end groups of the polymer and the anhydride functionalities of the chain extender [27].

In the nanocomposites, the quaternary ammonium salt used as the organic modifier decomposed via Hofmann elimination at the extrusion temperature, generating acidic sites on the silicate layers that catalyzed PC degradation [32,46]. Consequently, the thermal stability of the nanocomposites was lower than that of the neat polymer; for example, NC-E3 exhibited a T_d5_ of 413.9 °C compared with 428.1 °C for PC-E3. Like neat PC, repeated processing further reduced the thermal stability of the nanocomposites. The addition of the chain extender markedly enhanced the thermal stability of the reprocessed nanocomposite, increasing T_d5_ from 413.9 °C for NC-E3 to 421.0 °C for NC-E3/J and T_d10_ from 426.3 °C to 433.4 °C. Notably, these values for NC-E3/J exceeded those of NC-E2 (T_d5_ = 420.3 °C, T_d10_ = 432.6 °C), confirming the effectiveness of the chain extender in stabilizing reprocessed PC/nanoclay nanocomposites. The residual weight at 615 °C of the nanocomposites was higher than that of neat PC due to the presence of nanoclay.

## 4. Conclusions

In this study, the reprocessability of PC/organoclay nanocomposites was investigated under three sequential twin-screw extrusion cycles. A multi-anhydride functional chain extender was incorporated into the three-times extruded nanocomposites to compensate for thermo-mechanical degradation in the reprocessed PC nanocomposites and extend their applicability in sectors such as automotive, electrical–electronic, and construction. The intensified mechanical stresses associated with repeated extrusion cycles improved clay dispersion while suppressing re-agglomeration phenomena in the PC/organoclay systems. TEM observations revealed that reprocessing enhanced the delamination of silicate layers within the polymer matrix, resulting in predominantly intercalated and exfoliated structures, whereas the incorporation of the chain extender induced a higher degree of nanoclay exfoliation in the three-times extruded nanocomposite. Rheological analyses demonstrated that repeated extrusion cycles caused pronounced thermo-mechanical degradation in both neat PC and PC/organoclay nanocomposites. The chain extender exerted a significant influence on the viscoelastic properties of the reprocessed materials: the complex viscosity at 0.1 Hz of three-times extruded neat PC and the PC/organoclay nanocomposite increased by 610% and 248%, respectively, due to the relinking of degraded PC chains. Tensile strength of both neat PC and the nanocomposites exhibited a decreasing trend with increasing numbers of extrusion cycles. Although enhanced nanoclay dispersion within the PC matrix, thermo-mechanical degradation was the dominant mechanism, leading to a substantial reduction in tensile strength through molecular weight deterioration. Chain extension and branching reactions effectively restored the tensile strength of both neat PC and the PC/organoclay nanocomposite subjected to three extrusion cycles. Thermogravimetric analysis corroborated the rheological and tensile results, showing that the thermal stability of the samples decreased with increasing reprocessing cycles. This degradation effect was more pronounced in PC/organoclay nanocomposites due to the catalytic influence of the organomodifier present in the nanoclay. Notably, the incorporation of the chain extender led to a marked improvement in thermal stability, as evidenced by shifts in the decomposition temperatures toward higher values.

## Figures and Tables

**Table 1:** DSC and TGA data of samples.

Sample	T_g_ (°C)	T_d5_ (°C)	T_d10_ (°C)	Residue at 615 °C (%)
PC-E1	141.2	433.5	447.7	2.5
PC-E2	140	431.6	446.4	3.7
PC-E3	137.6	428.1	442.2	2.3
PC-E3/J	138.6	430.3	445.1	3.6
NC-E1	136.8	425.1	441.7	20.9
NC-E2	135.4	420.3	432.6	20.1
NC-E3	134.9	413.9	426.3	14.3
NC-E3/J	134.6	421	433.4	20.6

## Data Availability

The data that support the findings of this study are available from the corresponding author upon reasonable request.
